# Circ_0010729 knockdown protects cardiomyocytes against hypoxic dysfunction via miR-370-3p/TRAF6 axis

**DOI:** 10.17179/excli2020-2809

**Published:** 2020-11-11

**Authors:** Jingjing Zhang, Chuanyu Gao, Jing Zhang, Famin Ye

**Affiliations:** 1 Coronary Care Unit, Department of Cardiology, People's Hospital of Zhengzhou University, Zhengzhou City, Henan Procince, China; 2 Department of Cardiology, People's Hospital of Zhengzhou University, Zhengzhou City, Henan Procince, China

**Keywords:** circ_0010729, miR-370-3p, TRAF6, cardiomyocytes, hypoxia

## Abstract

Few studies have addressed the mechanism by which circ_0010729 regulates hypoxia-induced cell injury in cardiovascular diseases. However, its role and its regulatory mechanism in myocardial infarction remain to be explored. Cell viability, cycle, apoptosis, and migration were analyzed using cell counting kit-8 assay, flow cytometry, caspase-3 activity assay kit and transwell assay, respectively. Tumor necrosis factor-α (TNF-α), and interleukin-6 (IL-6) concentrations were examined by enzyme-linked immunosorbent assay. Glucose metabolism was calculated by detecting ATP production, glucose uptake and lactate production. Levels of circ_0010729, miR-370-3p and TNF Receptor Associated Factor 6 (TRAF6) were detected using quantitative real-time polymerase chain reaction or western blot. The direct interaction between circ_0010729 and TRAF6 or miR-370-3p was verified using dual-luciferase reporter assay and RNA immunoprecipitation assay. Under hypoxia condition, cardiomyocytes suffered from cell viability suppression, cell cycle arrest, cell apoptosis promotion, migration reduction, increase of inflammatory factor IL-6 and TNF-α, as well as glycolysis inhibition. Circ_0010729 expression was up-regulated in the cardiomyocytes at different hypoxia-exposed time points. Circ_0010729 knockdown protected cardiomyocytes against hypoxic dysfunction, while circ_0010729 overexpression showed inverse effects. MiR-370-3p was confirmed to directly bind to circ_0010729 or TRAF6. MiR-370-3p inhibition attenuated the protective effects of circ_0010729 knockdown on hypoxia-modulated cardiomyocyte dysfunction. MiR-370-3p restoration protected cardiomyocytes against hypoxic injury via targeting TRAF6. Besides, circ_0010729 indirectly regulated TRAF6 expression via miR-370-3p. This study demonstrated that circ_0010729 knockdown attenuated hypoxia-induced cardiomyocyte dysfunction via miR-370-3p/TRAF6 axis, indicating a potential therapeutic target for myocardial infarction.

## Introduction

Oxygen is a key micro-environmental substrate for sustaining tissue homeostasis in mammals, insufficient oxygen supply, or hypoxia is associated with diverse deadliest human diseases, including chronic obstructive pulmonary disease, stroke, cancer and coronary artery disease (Majmundar et al., 2010[16]; Serocki et al., 2018[20]). Acute myocardial infarction (AMI) is the leading pathological cause of disability and mortality in cardiovascular disease, which refers to cardiomyocyte dysfunction caused by myocardial ischemia and ischemia-associated hypoxia, eventually leads to heart failure (Anderson and Morrow, 2017[2]; Giordano, 2005[13]). Besides, cardiomyocytes are terminally differentiated cells without regenerative potentialities, thus, investigation of the mechanisms by which ischemia-associated hypoxia modulates cardiomyocytes dysfunction may be of great significance for the development of clinically therapeutic strategy of AMI.

Circular RNAs (circRNAs) are a subclass of endogenous noncoding RNAs made of covalently closed continuous loop structures, which render these molecules resist to RNase R decay (Ebbesen et al., 2016[9]). CircRNAs are often derived from exons, introns, or intergenic regions, high abundance along with the structural stability in eukaryotes and have tissue/cell-specific expression patterns (Qu et al., 2015[19]; Zhang et al., 2018[25]). CircRNAs are involved in almost all cellular processes, and accumulating evidence has shown that circ-RNAs play essential roles in the pathogenesis of multiple heart diseases, and have great potential as prognostic, diagnostic, and therapeutic biomarkers (Altesha et al., 2019[1]; Fan et al., 2017[10]; Qu et al., 2015[19]). Previous studies have found that hypoxia induced circ_0010729 up-regulation in human umbilical vein endothelial cells (HUVECs), and silencing circ_0010729 repressed the proliferative and migratory abilities and promoted apoptosis in hypoxia-induced HUVECs via down-regulating hypoxia inducible factor 1 alpha (HIF-1α) via microRNA (miR)-186 (Dang et al., 2017[7]). Additionally, Jin and Chen demonstrated that circ_0010729 was significantly elevated in oxygen-glucose-deprivation (OGD) condition, and strengthened OGD-evoked cell viability and migration reduction, and apoptosis promotion in human cardiomyocytes via regulating miR-145-5p (Jin and Chen, 2019[14]). Thus, we know that circ_0010729 is abnormally altered after hypoxia, while its role and its regulatory mechanism in myocardial infarction (MI) remain to be elucidated. 

Herein, this work focused on investigating the physiological role of circ_0010729 in cardiomyocyte phenotypic changes and glycolysis under hypoxia condition, and explored the potential regulatory network underlying circ_0010729 in hypoxia-induced cardiomyocyte dysfunction. 

## Materials and Methods

### Cell culture and low oxygen treatment

Human ventricular cardiomyocytes (AC-16) cells were obtained from Beijing Institute for Cancer Research Collection (Beijing, China) and cultured in Dulbecco's modified Eagle's medium/F-12 supplemented (DMEM/F12, Invitrogen, Waltham, MA, USA) supplemented with 12.5 % fetal bovine serum (FBS, Gibco, Carlsbad, CA, USA) and 1 % antibiotic-antimycotic (Gibco) with 5 % CO_2_ at 37 °C. Hypoxia was induced by exposing AC-16 cells to 1 % O_2_, 94 % N_2_, and 5 % CO_2_ for 12, 24, or 48 h using a modular incubator. Cells grown under a normoxic atmosphere (incubation with hypoxia condition for 0 h) were used as the control. 

### Cell transfection 

When cells were grown to 80 %-90 % confluency, 100 ng of circ_0010729 or TNF Receptor Associated Factor 6 (TRAF6) overexpression vector (circ or TRAF6) or nontarget plasmid (vector or pcDNA) (Promega, Madison, WI, USA), 50 nM of small interfering RNA (siRNA) against circ_0010729 (si-circ) or siRNA negative control (si-NC) (GenePharma, Shanghai, China), 40 nM of miR-370-3p mimic or miR-370-3p inhibitor (miR-370-3p, anti-miR-370-3p) or their negative control (miR-NC, anti-NC) (GenePharma) were transfected into AC-16 cells using Lipofectamine 3000 reagent (Invitrogen, Carlsbad, CA, USA).

### Cell counting kit-8 (cck-8) assay 

Following transfection and/or treatment, AC-16 cells (5000/well) were cultivated into a 96-well plate and co-interacted with 10 μL CCK-8 solution (Dojindo Molecular Technologies, Japan) at 37 °C for 4 h. The absorbance of each well was measured at 450 nm using a microplate reader. The results represented as the average of three independent replicates.

### Flow cytometer

After transfection and/or treatment, for cell cycle analysis, AC-16 cells were firstly digested by trypsin to obtain single-cell suspensions. After washing by PBS twice, cells were fixed by 75 % ethanol for 4 h at 4 °C, followed by incubation with 500 uL propidium iodide (PI) staining solution for 15 minutes. The quantitation of cell cycle distribution was analyzed using a FACScan flow cytometer (BD Biosciences, San Jose, CA, USA) with FlowJo software. For cell apoptosis analysis, AC-16 cells were harvested and washed in PBS, then double-stained with 10 μL of Annexin V-FITC and PI (BD Biosciences) for 15 min. Cell apoptosis was analyzed by the flow cytometer. All experiments were repeated three times independently. 

### Activity detection of caspase3

The activity of caspase3 was assessed using the commercial caspase-3 activity assay kit (Beyotime, Shanghai, China) following the guidance of producer. The activity was proportional to the absorbance which was detected at optical density (OD) 405 nm using the microplate reader. The results were represented as the average of three independent replicates. 

### Transwell assay

A transwell insert (Cell Biolabs, Inc. Santiago, CA, USA) without Matrigel (BD Biosciences) was employed to detect cell migration. Following transfection and/or treatment, AC-16 cells suspended in 200 μL serum-free medium were placed into the upper chamber of Transwell, then 600 μL medium fixed with FBS was added into the bottom chamber. After incubation for 24 h, migrated cells on the lower face of the chamber were counted by an inverted light microscope in five random fields (100 ×). Experiments were performed three times.

### Enzyme-linked immunosorbent assay (ELISA)

The concentrations of interleukin-6 (IL-6) and tumor necrosis factor-α (TNF-α) from the supernatants of AC-16 cells following appropriate transfection and/or treatment were determined using commercial IL-6 and TNF-α ELISA kits (R&D Systems, Minneapolis, Minnesota, USA) referring to the instructions of protocol. The results represented as the average of three independent replicates. 

### Glucose consumption and lactate production 

After transfection and/or treatment, the supernatants of AC-16 cell culture media were collected, and subjected to the analysis of the consumption or production of glucose and lactate using a Glucose Uptake Assay Kit and L-Lactate Assay Kit (Sigma, St Louis, MO, USA) referring to the producer's guidance using a microplate reader. Experiments were performed three times.

### Detection of ATP level

An ATP Assay Kit (Sigma) was applied to detect the level of ATP. Sonicated AC-16 cells were lysed, and the lysate was fixed with ATP reaction mix for 30 min. Finally, the OD570 nm value was examined using a microplate reader. Experiments were performed three times. 

### Quantitative real-time polymerase chain reaction (qRT-PCR)

Total RNA was extracted using Trizol reagent (Invitrogen) from cells. Then reverse transcription was performed using 1 μg of total RNA with a reverse transcription kit (Takara, Tokyo, Japan) to synthesize cDNA. Subsequently, qRT-PCR was conducted using SYBR Green PCR master mix (Takara) on an ABI 7500 Real-Time PCR system. The 2^−ΔΔCt^ method was used to calculate the fold changes with U6 or glyceraldehyde 3-phosphate dehydrogenase (GADPH) as an internal control. The same experiment was repeated three times, and the average was taken. The following primers were used: circ_0010729: F, 5'-CAGGCAGAGGTCCGGGCCTGTT-3' and R, 5'-GGACCGTTCTCAATGGCGTATAC-3'; GADPH: F, 5'-GGTGAAGGTCGGAGTCAAC-3' and R, 5'-AGAGTTAAAAGCAGCCCTGGTG-3'; TRAF6: F, 5'-CAGTGGTCGTATCGTGCTTA-3' and R, 5'-CCTTATGGT TTCTTGGAGTC-3'; miR-370-3p: F, 5'-GCCTGCTGGGGTGGAACCTGGT-3' and R, 5'-CTCAACTGGTGTCGTGGA -3'; U6: F, 5'-CTCGCTTCGGCAGCACA-3' and R, 5'-AACGCTTCACGAATTTGCGT-3'.

### Dual-luciferase reporter assay 

The sequences of circ_0010729 or TRAF6 3'UTR containing the wild-type or mutant potential binding sites of miR-370-3p were cloned into the pmirGLO luciferase vector (Promega), named wild-type/mutant-circ_0010729 or wild-type/mutant-3'UTR TRAF6. Then AC-16 cells placed on the 6-well plates were transfected with these constructed reporter plasmids and miR-370-3p or miR-NC using Lipofectamine 3000 (Invitrogen). Luciferase activities were determined using a dual-luciferase reporter assay kit (Promega). Each group was run in triplicate in 6-well plates. 

### RNA immunoprecipitation (RIP) assay

AC-16 cells were lysed using RIP buffer, and then incubated with RIPA buffer containing magnetic beads conjugated with human Anti-Ago2 antibody (Millipore, Billerica, MA, USA) or normal mouse Anti-IgG (Millipore). After interaction with Proteinase K, the immunoprecipitated RNA was extracted and purified RNA was determined using qRT-PCR. All experiments were repeated three times independently.

### Western blot

Proteins were extracted from cells using RIPA lysis buffer (Beyotime), and approximately 30 μg of extracted protein was subjected to western blot assay as described previously (Park et al., 2018[17]). The following antibodies were used: TRAF6 (1:2000, ab181622), and HRP-conjugated antibody (1:1000, ab9482), which all were obtained from Abcam (Cambridge, MA, USA). β-actin (1: 2000; #ZRB1312, Sigma) served as an internal control, and protein bands were visualized by a Super ECL assay kit (YRBIO, Changsha, Hunan, China). Triplicate individual experiments were performed in this study. 

### Statistical analysis

Data from thrice-repeated experiments were exhibited as mean ± standard deviation (SD). All quantitative data were analyzed using the Student's *t*-test, non-parametric test (Mann-Whitney U tests) (two groups) and one-way analysis of variance (ANOVA) (three or more groups). *P* values < 0.05 were considered statistically significant.

## Results

### Hypoxia triggers cardiomyocyte injury and glycolysis suppression

**Figure 1:**
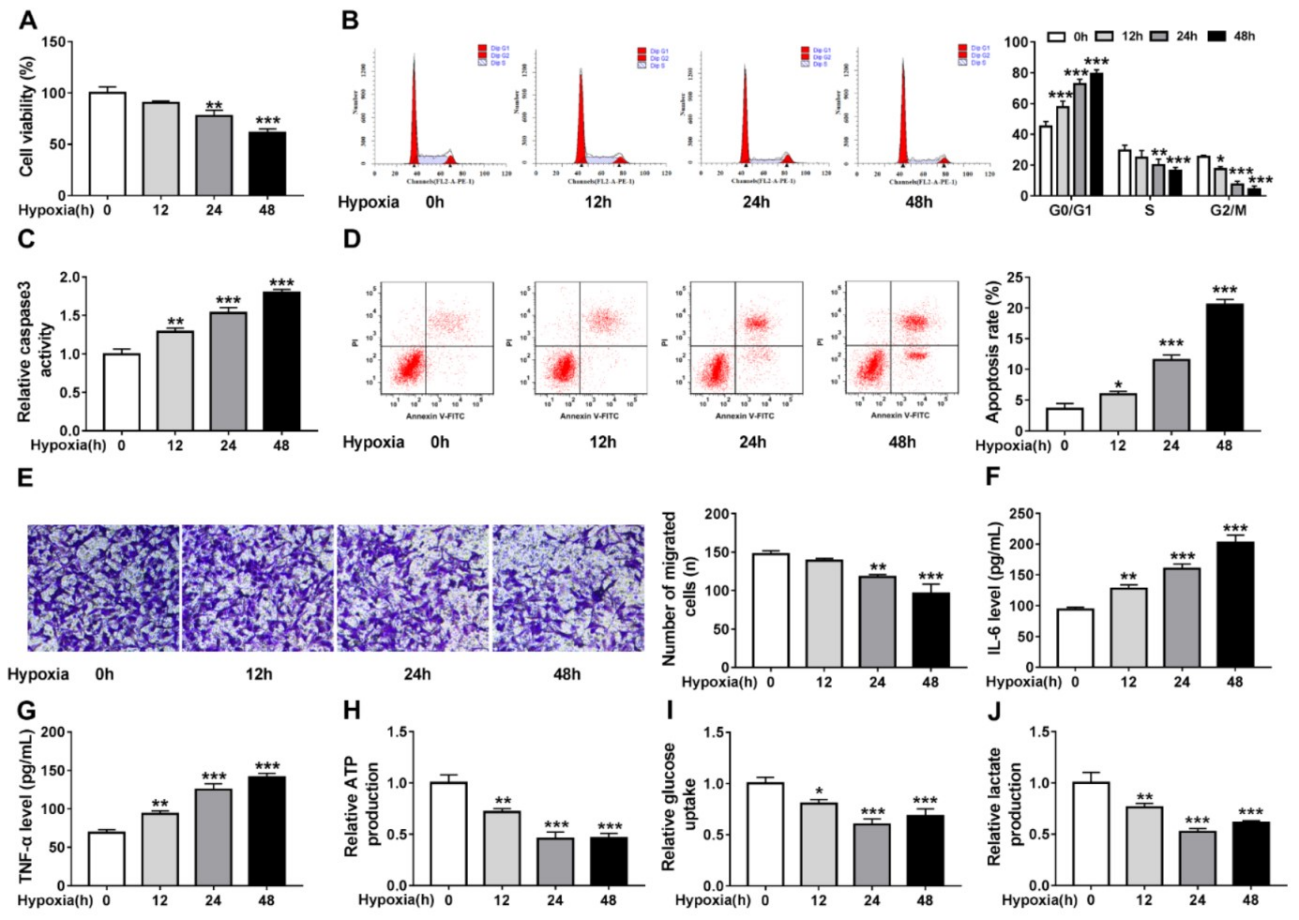
Hypoxia triggers cardiomyocyte injury and glycolysis suppression. Cardiomyocytes AC-16 were exposed to hypoxia for 0, 12, 24, and 48 h. (A) CCK-8 assay of cell viability analysis. (B) Cell cycle analysis using flow cytometry. (C) Detection of caspase3 activity in cells using a colorimetric assay kit. (D) Apoptosis analysis of cells using flow cytometry. (E) Transwell assay of cell migration. (F, G) Levels' detection of IL-6 and TNF-α using ELISA assay. (H-J) Measurement of ATP production, glucose uptake and lactate production using the colorimetric assay kits. *P<0.05, **P<0.01, ***P<0.001, ****P<0.0001.

First, the effects of hypoxia on cardiomyocyte properties were tested. Cardiomyocyte AC-16 cells were exposed to hypoxia for 0, 12, 24, and 48 h, by contrast with the control (0 h) group, hypoxia led to AC-16 cell viability suppression (Figure 1A(Fig. 1)), cell cycle arrest (Figure 1B(Fig. 1)), caspase3 activity enhancement (Figure 1C(Fig. 1)), cell apoptosis promotion (Figure 1D(Fig. 1)), as well as cell migration inhibition (Figure 1E(Fig. 1)). Besides, the levels of inflammatory factor IL-6 and TNF-α were found to be significantly increased under hypoxia at 12, 24, or 48 h (Figure 1F, G(Fig. 1)). Results in Figure 1H-J(Fig. 1) exhibited hypoxia suppressed glycolysis in AC-16 cells, reflected by the decrease of ATP production (Figure 1H(Fig. 1)), glucose uptake (Figure 1I(Fig. 1)) and lactate production (Figure 1J(Fig. 1)) at 12, 24 and 48 h exposure. These results suggested that hypoxia-induced cardiomyocyte injury and suppressed glycolysis.

### Circ_0010729 knockdown reverses hypoxia-induced cardiomyocyte injury and glycolysis suppression 

**Figure 2:**
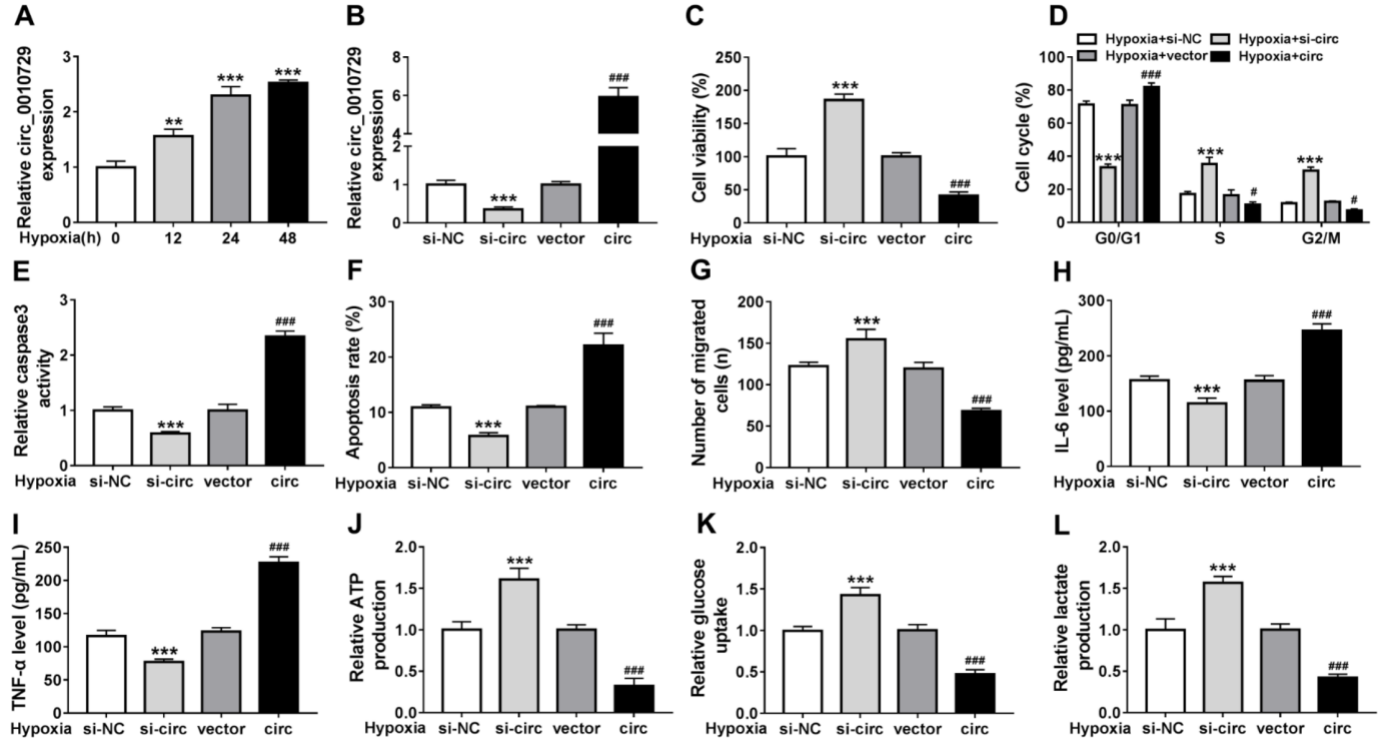
Circ_0010729 knockdown reverses hypoxia-induced cardiomyocyte injury and glycolysis suppression. (A) qRT-PCR analysis of circ_0010729 expression in AC-16 cells after treatment with hypoxia at 12, 24, and 48 h. (B) qRT-PCR analysis of circ_0010729 expression in AC-16 cells transfected with vector, circ_0010729 (circ), si-NC or si-circ_0010729 (si-circ). After treatment with hypoxia for 24 h, (C) CCK-8 assay of cell viability analysis; (D) flow cytometry of cell cycle; (E) caspase3 activity analysis in cells using a colorimetric assay kit; (F) cell apoptosis analysis using flow cytometry; (G) transwell assay of cell migration. (H, I) Levels' detection of IL-6 and TNF-α using ELISA assay. (J-L) Measurement of ATP production, glucose uptake and lactate production using the colorimetric assay kits. **P<0.01, ***P<0.001,^ #^*P*<0.05, ^###^*P*<0.001.

The molecular mechanism of hypoxia-modulated injury and glycolysis in cardiomyocytes was then investigated. We found circ_0010729 was elevated by hypoxia exposure at 12, 24, and 48 h (Figure 2A(Fig. 2)). To investigate whether the promotion of the hypoxia-induced circ_0010729 could protect cardiomyocytes in hypoxia conditions, AC-16 cells were transfected with circ_0010729 (circ) or specific si-circ_0010729 (si-circ). As expected, circ_0010729 expression was markedly up-regulated in AC-16 cells when transfected with circ_0010729, while circ_0010729 expression was down-regulated by si-circ_0010729 compared with their counterparts, respectively (Figure 2B(Fig. 2)). After treatment with hypoxia for 24 h, circ_0010729 knockdown promoted cell viability (Figure 2C(Fig. 2)) and cell cycle progression (Figure 2D(Fig. 2)), suppressed caspase3 activity (Figure 2E(Fig. 2)), apoptosis (Figure 2F(Fig. 2)), and migration (Figure 2G(Fig. 2)), reduced IL-6 and TNF-α release (Figure 2H, I(Fig. 2)), as well as enhanced ATP production (Figure 2J(Fig. 2)), glucose uptake (Figure 2K(Fig. 2)) and lactate production (Figure 2L(Fig. 2)) in hypoxia-treated AC-16 cells, while the introduction of circ_0010729 in AC-16 cells exhibited inverse effects (Figure 2C-L(Fig. 2)). Taken together, knockdown of circ_0010729 might protect cardiomyocytes through restoration of cardiomyocyte properties and glucose metabolism. 

### miR-370-3p is a target of circ_0010729

**Figure 3:**
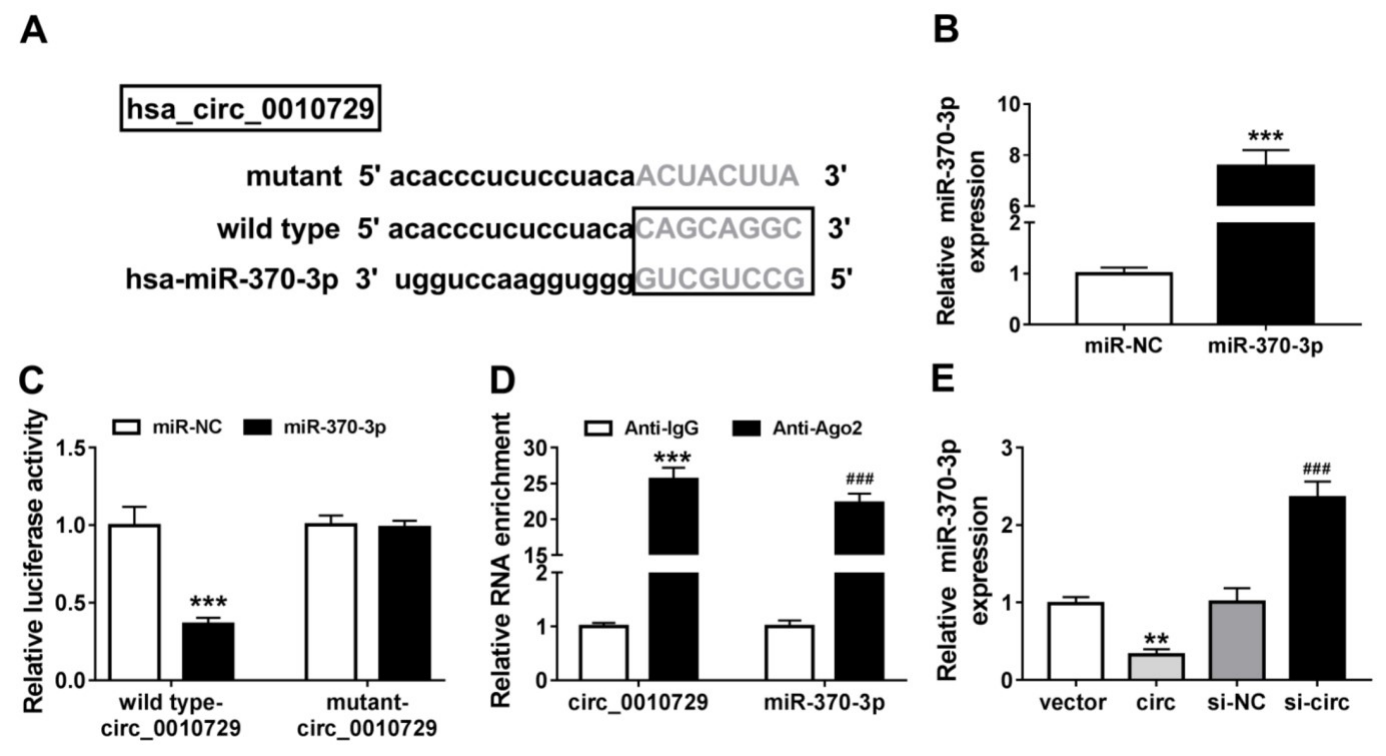
MiR-370-3p is a target of circ_0010729. (A) The putative binding sequences of miR-370-3p on circ_0010729. (B) qRT-PCR analysis of miR-370-3p expression in AC-16 cells transfected with miR-370-3p or miR-NC. (C) Dual-luciferase reporter assay in AC-16 cells co-transfected with wild type-circ_0010729 or mutant-circ_0010729 and the indicated miRNAs. (D) RIP assay for the enrichment of Ago2 on miR-370-3p and circ_0010729 in AC-16 cells. (E) qRT-PCR analysis of miR-370-3p expression in AC-16 cells transfected with vector, circ_0010729 (circ), si-NC or si-circ_0010729 (si-circ). **P<0.01, ***P<0.001, ^###^*P*<0.001.

To explore molecular mechanism underlying the action of circ_0010729 in hypoxia-modulated cardiomyocyte properties, the online database CircInteractome was applied to predict the potential microRNA (miRNA) that could be interacted with circ_0010729. Then miR-370-3p was identified to have the potential binding sites of circ_0010729 (Figure 3A(Fig. 3)). Afterwards, the transfection efficiency of miR-370-3p or miR-NC was validated, as expected, miR-370-3p expression was greatly overexpressed in AC-16 cells after miR-370-3p transfection (Figure 3B(Fig. 3)). Immediately, the dual-luciferase reporter assay showed miR-370-3p overexpression significantly reduced the luciferase activity in AC-16 cells transfected with wild type-circ_0010729 (Figure 3C(Fig. 3)). Meanwhile, data from RIP assay revealed that circ_0010729 and miR-370-3p were highly enriched in the complex precipitated by Anti-Ago2 compared with nonspecific Anti-IgG (Figure 3D(Fig. 3)). Importantly, the effect of circ_0010729 on miR-370-3p expression was investigated, qRT-PCR analysis indicated miR-370-3p expression in AC-16 cells was decreased by circ_0010729 overexpression, but increased by circ_0010729 down-regulation (Figure 3E(Fig. 3)). Altogether, circ_0010729 directly bound to miR-370-3p and negatively regulated its expression.

### Knockdown of circ_0010729 protects cardiomyocytes against hypoxia-induced injury through miR-370-3p

**Figure 4:**
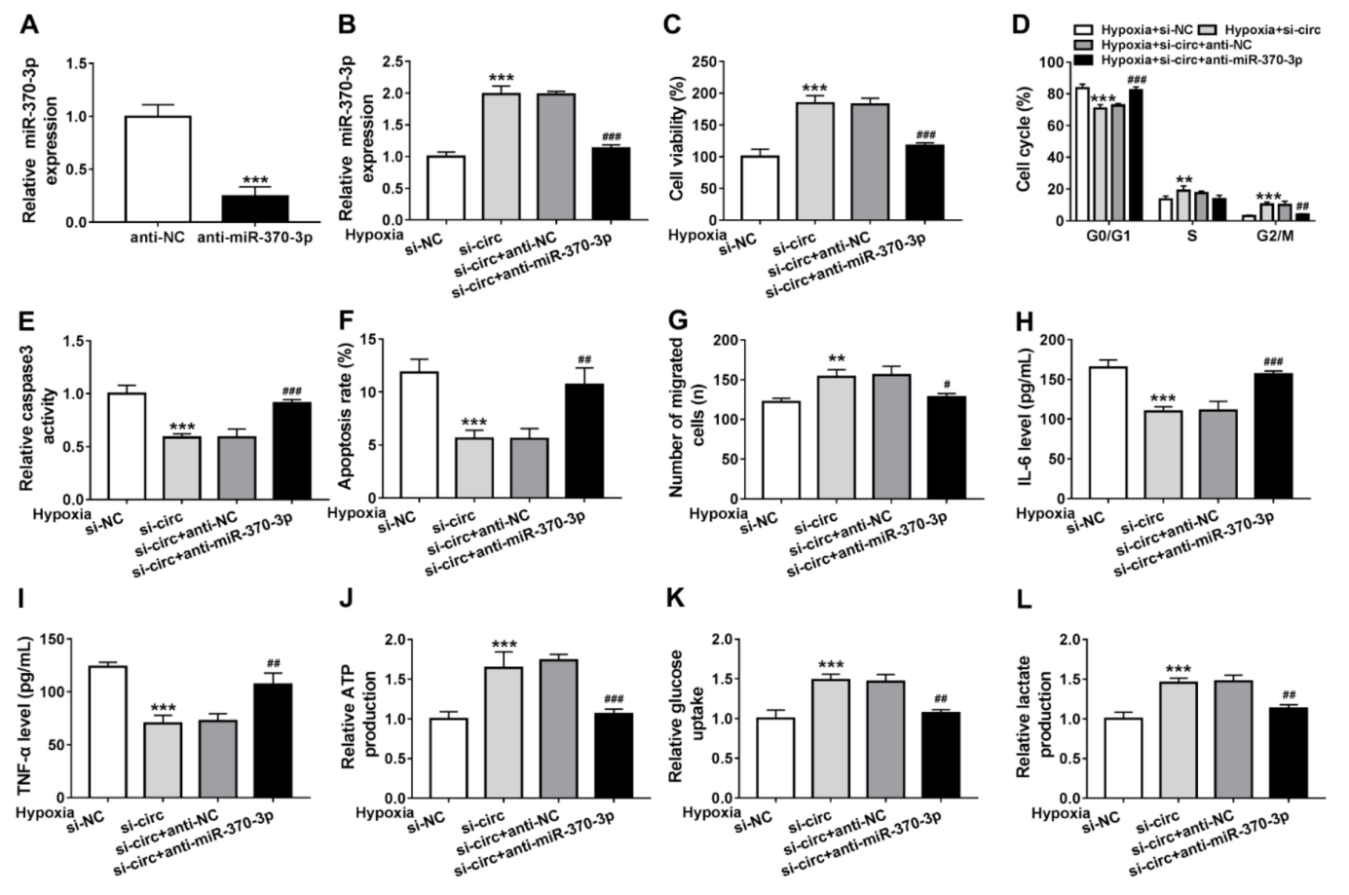
Knockdown of circ_0010729 protects cardiomyocytes against hypoxia-induced injury through miR-370-3p. (A) qRT-PCR analysis of miR-370-3p expression in AC-16 cells transfected with anti-NC or anti-miR-370-3p. (B) qRT-PCR analysis of miR-370-3p expression in AC-16 cells transfected with si-NC, si-circ_0010729 (si-circ), si-circ_0010729 (si-circ) + anti-NC, or si-circ_0010729 (si-circ) + anti-miR-3701-3p under hypoxia for 24 h. (C) CCK-8 assay of cell viability analysis. (D) Cell cycle analysis using flow cytometry. (E) Analysis of caspase3 activity in cells using a colorimetric assay kit. (F) Apoptosis analysis of cells using flow cytometry. (G) Transwell assay of cell migration. (H, I) Levels' detection of IL-6 and TNF-α using ELISA assay. (J-L) Measurement of ATP production, glucose uptake and lactate production using the colorimetric assay kits. **P<0.01, ***P<0.001,^ #^*P*<0.05, ^##^*P*<0.01 ^###^*P*<0.001.

Based on the relation between circ_0010729 and miR-370-3p, we then investigated whether circ_0010729 regulated cardiomyocytes properties under hypoxia was through binding to miR-370-3p. First of all, AC-16 cells were transfected with anti-miR-370-3p or anti-NC, as expected, anti-miR-370-3p introduction caused significant reduction of miR-370-3p expression relative to anti-NC (Figure 4A(Fig. 4)). Next, we transfected anti-miR-370-3p into circ_0010729-decreased AC-16 cells, and found the introduction of anti-miR-370-3p attenuated circ_0010729 knockdown-induced miR-370-3p overexpression in hypoxia condition (Figure 4B(Fig. 4)). Then under hypoxia for 24 h, we found miR-370-3p inhibitor reversed the regulatory effects of si-circ_0010729 on AC-16 cell viability (Figure 4C(Fig. 4)), cell cycle (Figure 4D(Fig. 4)), caspase3 activity (Figure 4E(Fig. 4)), apoptosis (Figure 4F(Fig. 4)), migration (Figure 4G(Fig. 4)), IL-6 and TNF-α release (Figure 4H, I(Fig. 4)) as well as glucose metabolism (Figure 4J-L(Fig. 4)). Altogether, knockdown of circ_0010729 might protect cardiomyocytes against hypoxia-induced injury through the restoration of cardiomyocytes' properties and glycolysis via miR-370-3p.

### TRAF6 is a target of miR-370-3p

**Figure 5:**
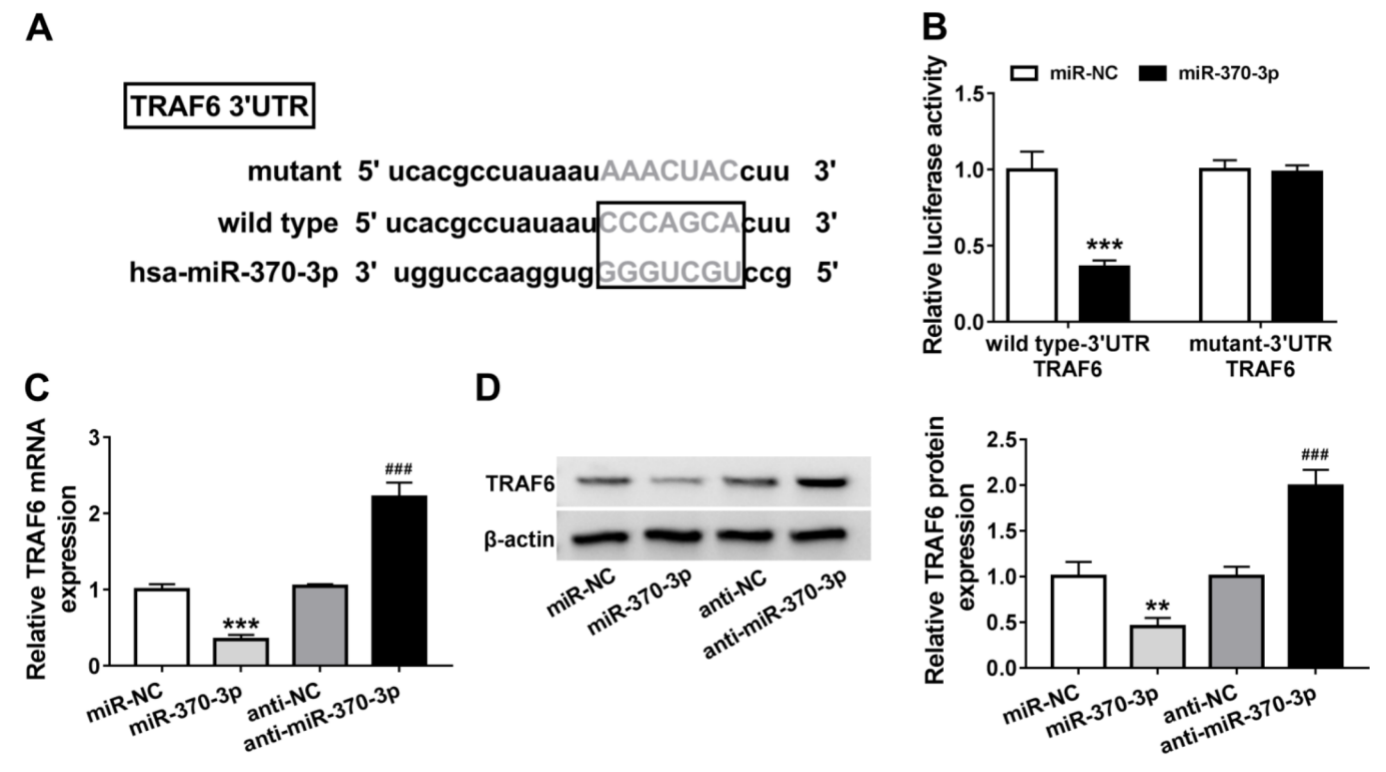
TRAF6 is a target of miR-370-3p. (A) The putative binding sequences of miR-370-3p on TRAF6. (B) Dual-luciferase reporter assay in AC-16 cells co-transfected with wild type-3' UTR TRAF6 or mutant-3' UTR TRAF6 and the indicated miRNAs. (C, D) qRT-PCR and western blot analysis of TRAF6 expression in AC-16 cells transfected with miR-370-3p, miR-NC, anti-NC, or anti-miR-370-3p. **P<0.01, ***P<0.001, ^###^*P*<0.001.

The downstream target genes of miR-370-3p were then explored. Through searching online database Targetscan, TRAF6 was identified as a potential target of miR-370-3p (Figure 5A(Fig. 5)). Then the significant reduction of luciferase activity in AC-16 cells co-transfected with wild-type-3'UTR TRAF6 and miR-370-3p confirmed their direct interaction (Figure 5B(Fig. 5)). After that, the effect of miR-370-3p on TRAF6 expression was detected, and we found TRAF6 expression both at mRNA and protein levels was decreased by miR-370-3p up-regulation, but increased by miR-370-3p down-regulation in AC-16 cells (Figure 5C, D(Fig. 5)). Thus, we confirmed miR-370-3p targetedly suppressed TRAF6. 

### Restoration of miR-370-3p protects cardiomyocytes against hypoxia-induced injury through TRAF6

**Figure 6:**
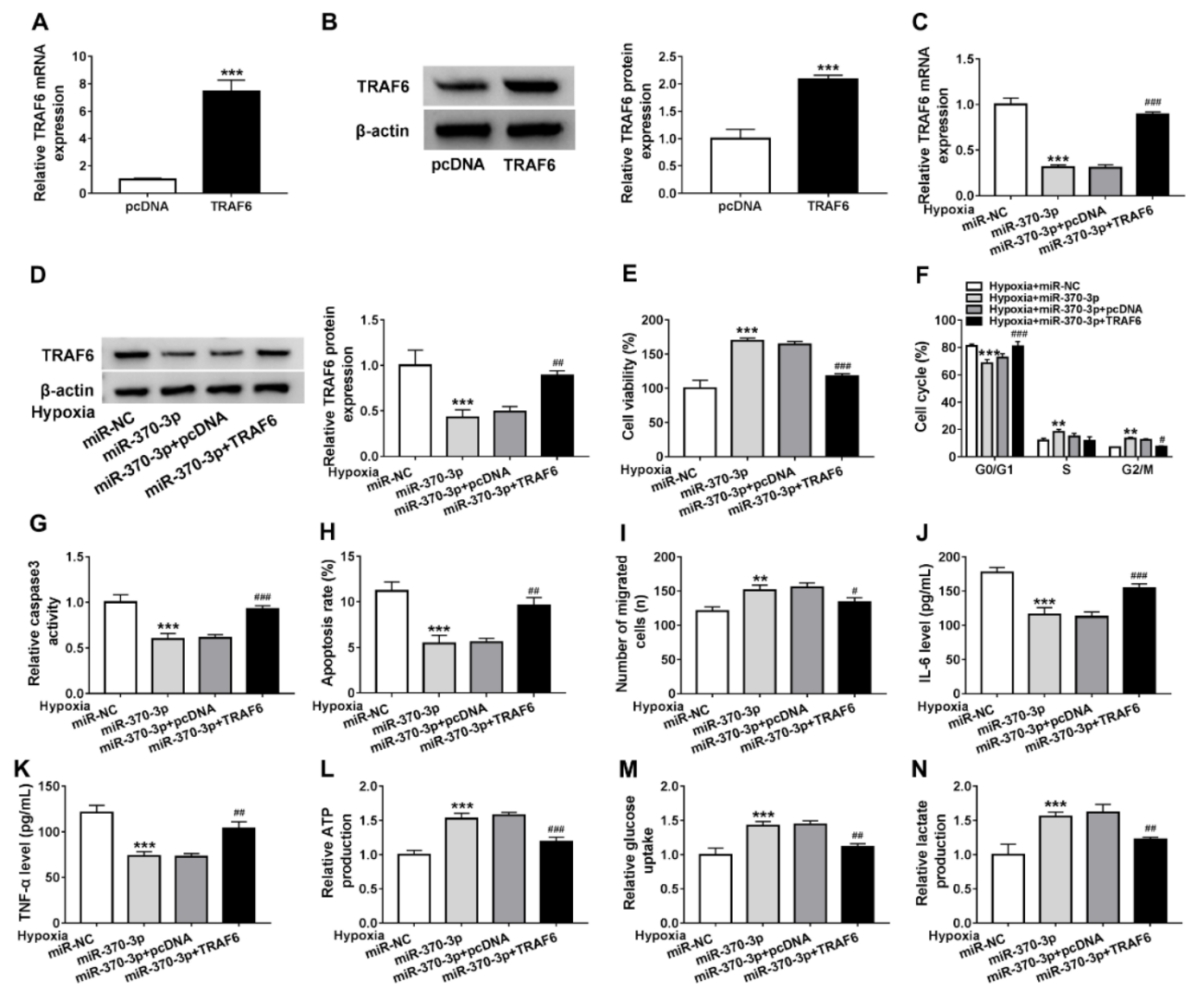
Restoration of miR-370-3p protects cardiomyocytes against hypoxia-induced injury through TRAF6. (A, B) qRT-PCR and western blot analysis of TRAF6 expression in AC-16 cells transfected with pcDNA or TRAF6. (C, D) qRT-PCR and western blot analysis of TRAF6 expression in AC-16 cells transfected with miR-NC, miR-370-3p, miR-370-3p + pcDNA, or miR-370-3p + TRAF6 under hypoxia for 24 h. (E) Cell viability analysis using CCK-8 assay. (F) Flow cytometry of cell cycle analysis. (G) Analysis of caspase3 activity in cells using a colorimetric assay kit. (H) Apoptosis analysis of cells using flow cytometry. (I) Cell migration analysis using transwell assay. (J, K) Levels' detection of IL-6 and TNF-α using ELISA assay. (L-N) Detection of ATP production, glucose uptake and lactate production using the colorimetric assay kits. **P<0.01, ***P<0.001, ^#^*P*<0.05, ^##^*P*<0.01 ^###^*P*<0.001.

Given the direct interaction between miR-370-3p and TRAF6, we then studied the functions miR-370-3p/TRAF6 axis on cardiomyocytes. First, AC-16 cells were transfected with pcDNA or TRAF6, and TRAF6 expression was markedly elevated in cells after TRAF6 transfection compared to pcDNA (Figure 6A, B(Fig. 6)). Next, AC-16 cells were co-transfected with miR-NC, miR-370-3p, miR-370-3p + pcDNA, or miR-370-3p + TRAF6, and we found that introduction of TRAF6 markedly rescued miR-370-3p-induced decrease of TRAF6 level in AC-16 cells (Figure 6C, D(Fig. 6)). Thereafter, transfected cells were exposed to hypoxia condition for 24 h and rescue assay was then performed. Results showed miR-370-3p re-expression attenuated hypoxia-induced AC-16 cell viability suppression (Figure 6E(Fig. 6)), cell cycle arrest (Figure 6F(Fig. 6)), caspase3 activity enhancement (Figure 6G(Fig. 6)), apoptosis promotion (Figure 6H(Fig. 6)), migration inhibition (Figure I(Fig. 6)), IL-6 and TNF-α levels increase (Figure J, K(Fig. 6)), and glycolysis suppression (Figure 6L-N(Fig. 6)), while these conditions were reversed by following TRAF6 overexpression (Figure 6E-N(Fig. 6)). Overall, miR-370-3p might protect cardiomyocytes through restoration of cardiomyocyte properties and glucose metabolism via TRAF6 in hypoxia condition.

### circ_0010729 regulates TRAF6 via binding to miR-370-3p

**Figure 7:**
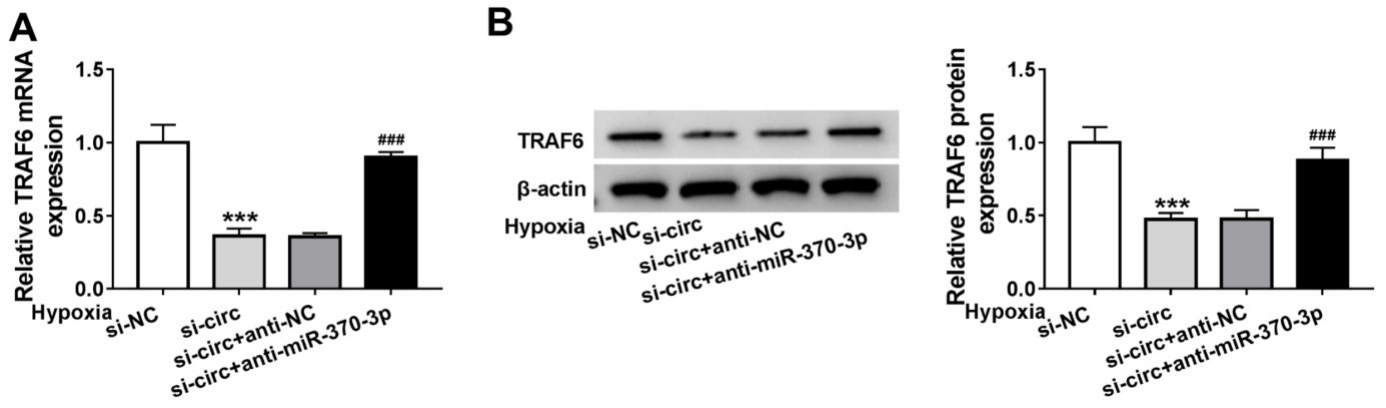
Circ_0010729 regulates TRAF6 via binding to miR-370-3p. (A, B) qRT-PCR and western blot analysis of TRAF6 expression in AC-16 cells transfected with si-NC, si-circ_0010729 (si-circ), si-circ_0010729 (si-circ) + anti-NC, or si-circ_0010729 (si-circ) + anti-miR-3701-3p under hypoxia for 24 h. **P<0.01, ***P<0.001, ^#^*P*<0.05, ^##^*P*<0.01 ^###^*P*<0.001.

Whether specific crosstalk existed between circ_0010729 and TRAF6 through competition for miR-370-3p binding was further investigated. As shown in Figure 7A, B(Fig. 7), we found miR-370-3p inhibition rescued circ_0010729 decrease-induced TRAF6 down-regulation under hypoxia condition. Thus, we confirmed that circ_0010729 could indirectly regulate TRAF6 via miR-370-3p. 

See also Supplementary data.

## Discussion

Currently, circRNAs have frequently been reported in cardiovascular disease and have important roles in ischemic heart diseases by regulating cellular biological processes (Altesha et al., 2019[1]). For example, Li et al. found circNCX1 was elevated in excessive reactive oxygen species (ROS) condition and enhanced ROS-induced cardiomyocyte apoptosis via regulating miR-133a-3p/ CDIP1, thus leading to ischemia-reperfusion damage (Li et al., 2018[15]). CircRNA Cdr1as strengthened hypoxia-stimulated cardiomyocyte apoptosis through absorbing miR-7a to aggravate MI (Geng et al., 2016[12]). Further evidence revealed that circFndc3b was down-regulated in cardiomyocytes, and restoration of its expression promoted cardiac function and remodeling after MI through inhibiting cardiomyocyte apoptosis and evoking neovascularization via FUS/VEGF-A axis (Garikipati et al., 2019[11]). Thus, circRNAs may be potential candidates for future therapeutic interventions in MI through regulating cardiomyocyte phenotypes. 

In this study, cardiomyocytes were exposed to hypoxic condition, and we found hypoxia triggered cell viability and migration suppression, cell cycle arrest, cell apoptosis promotion, as well as increase of inflammatory factor IL-6 and TNF-α, thus resulting in ischemic cardiomyocytes' dysfunction. Besides, it is reported that metabolic changes occur in the myocardium during ischemia-associated hypoxia due to the deprivation of oxygen and nutrient supply (Zhang et al., 2017[24]). Therefore, we also found hypoxia induced glycolysis suppression, evidenced by the reduction of ATP production, glucose uptake and lactate production. Then we found circ_0010729 was increased in hypoxia-treated cardiomyocytes, and hypoxia-evoked cardiomyocyte phenotypic changes and glycolysis suppression were attenuated when circ_0010729 was down-regulated, while overexpressed circ_0010729 in cardiomyocytes showed inverse effects. Taken together, down-regulation of circ_0010729 exhibited cardioprotective effects by reducing hypoxia-evoked cardiomyocyte dysfunction. 

In mechanism, circRNAs have been widely reported as efficient miRNA “sponges” with gene-regulatory potential (Chen et al., 2017[5]; Du et al., 2017[8]). Current studies indicated that circRNAs contain at least one miRNA binding site (Thomas and Sætrom, 2014[21]).Thus, we identified miRNAs which might interact with circ_0010729 by using bioinformatics tools, and confirmed that miR-370-3p was the downstream target of circ_0010729 in cardiomyocytes. Micro-RNAs are vital epigenetic regulatory molecules, and increasing evidence has exhibited the prominent roles of them in progression and development of MI (Chistiakov et al., 2016[6]). A mass of miRNAs exhibited abnormally altered in the pathological process of MI, and miRNAs have potential as therapeutic biomarkers of MI (Bejerano et al., 2018[3]; Boon and Dimmeler, 2015[4]). For instance, miR-27a-5p alleviated hypoxia-evoked rat cardiomyocyte damage via modulating autophagy and apoptosis through Atg7 (Zhang et al., 2019[23]). MiR-21 attenuated hypoxia-induced cardiomyocyte apoptosis by suppressing PTEN expression (Wu et al., 2019[22]). Additionally, miR-370 was demonstrated to have cardioprotective effects on hypoxia-induced cardiomyocyte injury via regulating cell oxidative stress and survival, which might be a new therapeutic target for MI (Qiu et al., 2019[18]; Zhao et al., 2019[26]). However, the role and mechanism of miR-370-3p in MI remain vague. 

In our work, we demonstrated that miR-370-3p overexpression antagonized hypoxia-induced cardiomyocyte phenotypic changes and glycolysis suppression; importantly, inhibition of miR-370-3p attenuated the protective effects of si-circ_0010729 on cardiomyocyte under hypoxia condition. Besides that, this study also confirmed that TRAF6 was a target of miR-370-3p, and was reduced by miR-370-3p overexpression. What's more, miR-370-3p restoration protected cardiomyocytes against hypoxia injury through TRAF6, and circ_0010729 could indirectly regulate TRAF6 via serving as a sponge of miR-370-3p in cardiomyocytes. Thus, the circ_0010729/miR-370-3p/TRAF6 regulatory network was identified in ischemia-associated hypoxia-induced cardiomyocytes dysfunction. 

In summary, our work suggested that knockdown of circ_0010729 weakened hypoxia-induced cardiomyocyte dysfunction via miR-370-3p/TRAF6 axis, suggesting a potential therapeutic target for protecting against cardiomyocyte dysfunction during hypoxia injury.

## Acknowledgement

None.

## Disclosure of interest

The authors declare that they have no financial conflicts of interest.

## Funding

This work was supported by Chengdu Municipal Health Commission Project (No. 2019107).

## Supplementary Material

Supplementary data
