# Supplementary material for: Circ_0010729 knockdown protects cardiomyocytes against hypoxic dysfunction via miR-370-3p/TRAF6 axis
Source: EXCLI J. 2020 Nov 11;19:1520–32. doi: 10.17179/excli2020-2809 (PMC7689242; doi:10.17179/excli2020-2809)
Supplement: Supplementary data [file EXCLI-19-1520-s-001.pdf]

EXCLI Journal 2020;19:1520-1532 – ISSN 1611-2156
Received: August 19, 2020, accepted: October 21, 2020, published: November 11, 2020

Supplementary data to:

circ_0010729 KNOCKDOWN PROTECTS CARDIOMYOCYTES AGAINST HYPOXIC DYSFUNCTION VIA miR-370-3p/TRAF6 AXIS

Jingjing Zhang[1], Chuanyu Gao[2,*], Jing Zhang[1], Famin Ye[1]

[1] Coronary Care Unit, Department of Cardiology, People's Hospital of Zhengzhou University, Zhengzhou City, Henan Procince, China
[2] Department of Cardiology, People's Hospital of Zhengzhou University, Zhengzhou City, Henan Procince, China

[*] **Corresponding author:** Chuanyu Gao, MD, Department of Cardiology, People's Hospital of Zhengzhou University, No.7 Weiwu Road, Jinshui District, Zhengzhou 450003, Henan Procince, China; Tel: +86 0371-58680639, Fax: +86 0371-58680639;
E-mail: gaochuanyuz@163.com

http://dx.doi.org/10.17179/excli2020-2809

This is an Open Access article distributed under the terms of the Creative Commons Attribution License (http://creativecommons.org/licenses/by/4.0/).

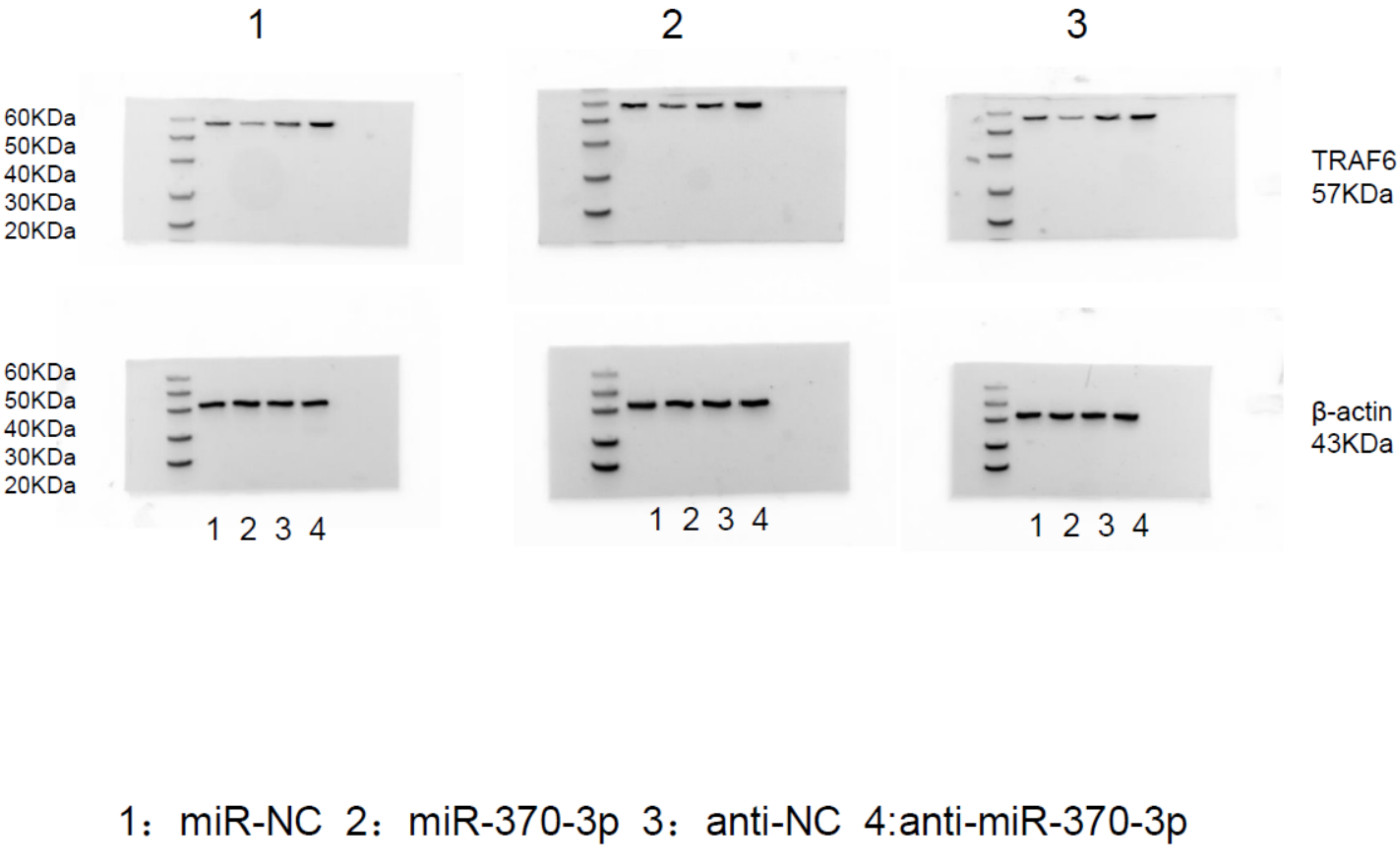
1
2
3
60KDa
50KDa
40KDa
30KDa
20KDa
TRAF6
57KDa
60KDa
50KDa
40KDa
30KDa
20KDa
β-actin
43KDa
1 2 3 4
1 2 3 4
1 2 3 4
1： miR-NC 2： miR-370-3p 3： anti-NC 4:anti-miR-370-3p

Figure 5D

EXCLI Journal 2020;19:1520-1532 – ISSN 1611-2156
Received: August 19, 2020, accepted: October 21, 2020, published: November 11, 2020

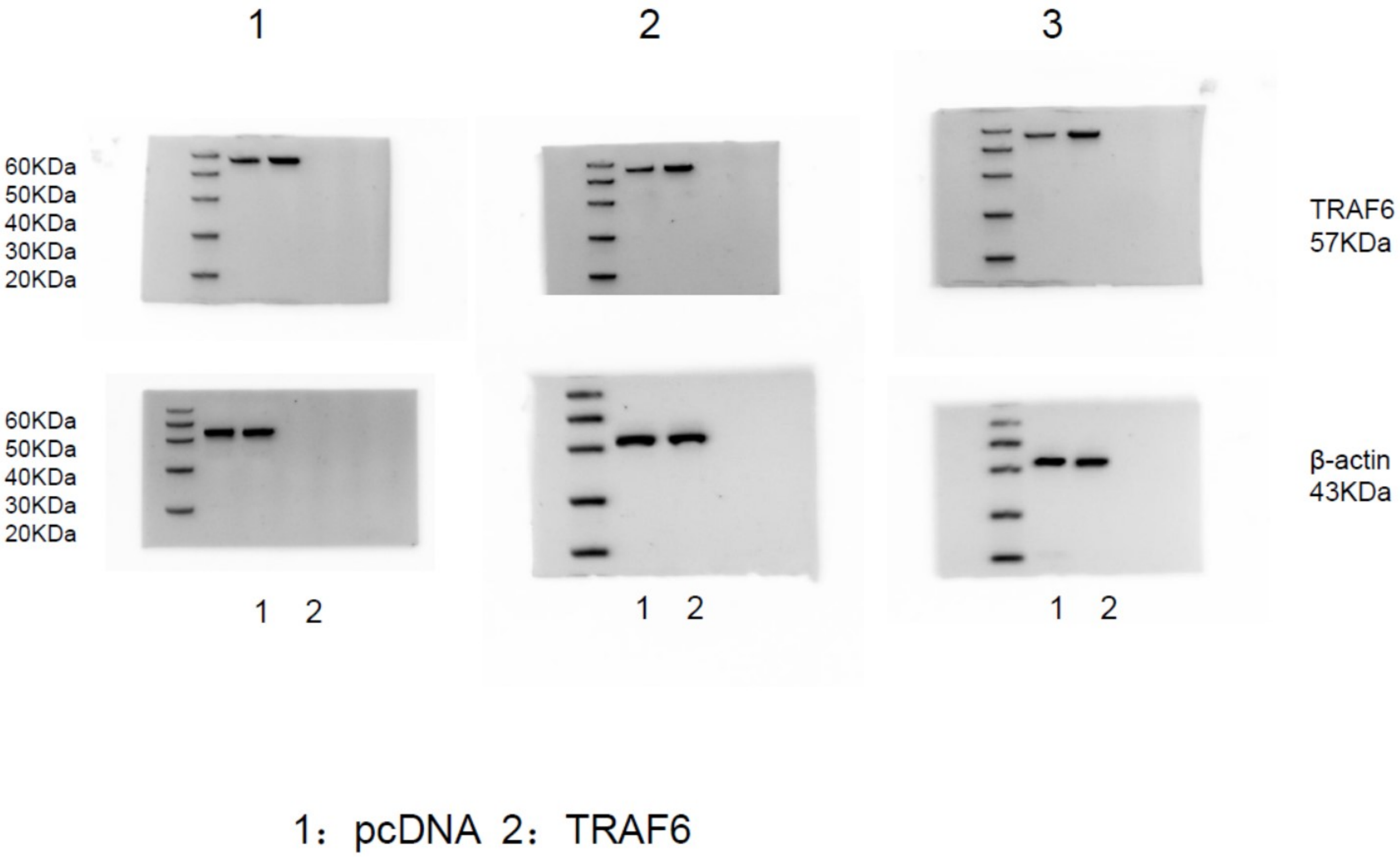

Figure 6B

EXCLI Journal 2020;19:1520-1532 – ISSN 1611-2156
Received: August 19, 2020, accepted: October 21, 2020, published: November 11, 2020

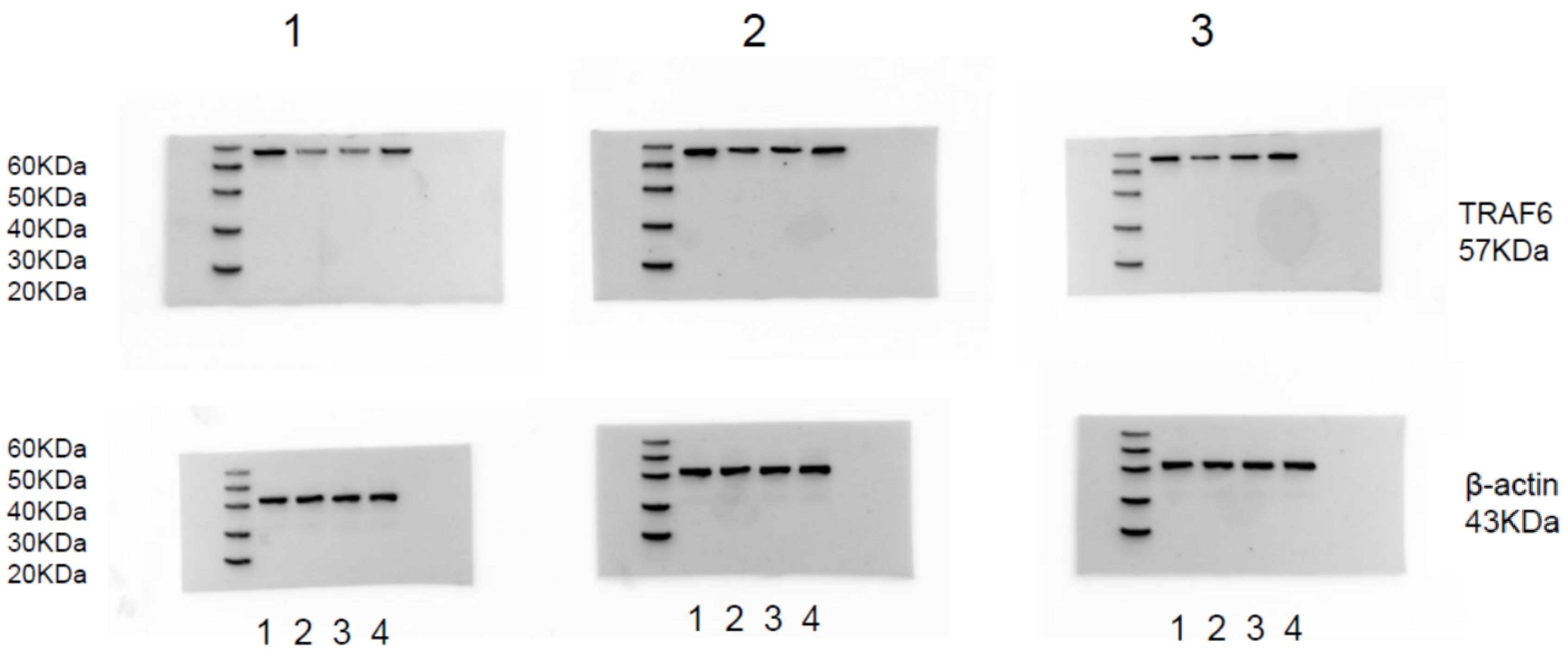

1： miR-NC 2： miR-370-3p 3： miR-370-3p+pcDNA 4： miR-370-3p+TRAF6

Figure 6D

EXCLI Journal 2020;19:1520-1532 – ISSN 1611-2156
Received: August 19, 2020, accepted: October 21, 2020, published: November 11, 2020

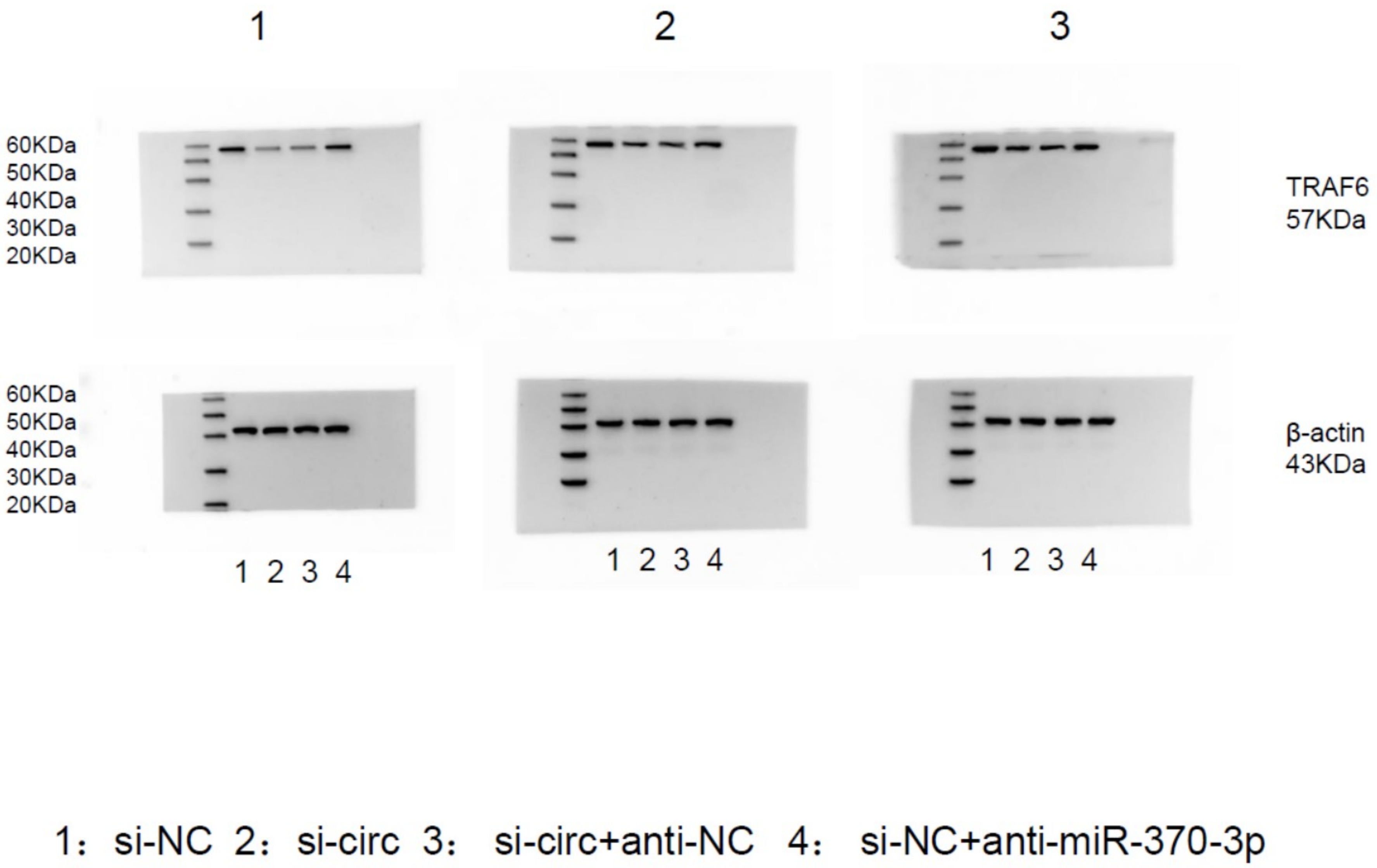

Figure 7B
